# Effects of perspective switching and utilitarian thinking on moral judgments in a sacrificial dilemma among healthcare and non-healthcare students

**DOI:** 10.1007/s12144-023-04380-z

**Published:** 2023-02-16

**Authors:** Junsu Park, Yongmin Shin, Seungmin Kim, Seho Maeng, Jungjoon Ihm

**Affiliations:** 1 Department of Social Entrepreneurship and Humanistic Future Studies, SungKyunKwan University, 25-2 Sungkyunkwan-Ro, Jongno-Gu, Seoul, 03063 South Korea; 2 Dental Research Institute, Seoul National University School of Dentistry, 101 Daehak-Ro, Jongno-Gu, Seoul, 03080 South Korea; 3 Graduate School of Counseling, The Catholic University of Korea, 43 Jibong-Ro, Bucheon, 14662 South Korea

**Keywords:** Utilitarian judgments, Instrumental harm, Impartial beneficence, Healthcare and non-healthcare students

## Abstract

During the COVID-19 pandemic, healthcare professionals have often faced moral challenges, which required them to choose between endorsing self- or other-sacrifice for the greater good. Drawing on the altruistic rationalization hypothesis and trait-activation theory, this study investigates (a) whether healthcare students’ endorsement of utilitarian solutions to sacrificial moral dilemmas varies when they are confronted with the minority group, majority group, or third-person perspective on the given dilemma and (b) whether individual differences in utilitarian thinking, as measured by the Oxford Utilitarianism Scale (both instrumental harm and impartial beneficence), predict endorsement of utilitarian solutions to moral dilemmas. The study population was divided into a group of healthcare students and a group of non-healthcare students. It was found that the members of both groups expressed a stronger pro-utilitarian position when making moral dilemma judgments from a majority perspective than from the two other perspectives. However, a difference was observed with healthcare students being more reluctant to endorse the utilitarian action than their non-healthcare counterparts in the *self-in-majority* context. The instrumental harm component was a significant predictor of utilitarian judgments in the healthcare group, but impartial beneficence significantly predicted utilitarian judgments in the non-healthcare group in the *self-in-majority* context.

**Supplementary Information:**

The online version contains supplementary material available at 10.1007/s12144-023-04380-z.

The COVID-19 pandemic has caused significant changes in social structures and community practices worldwide, and these have had impacts on individual moral principles, reasoning, and judgment (Francis & McNabb, 2022). This has resulted in strain being placed on the morality of healthcare professionals. They are at a high risk of being infected with COVID-19 at their place of employment, which requires them to face the serious moral dilemma of having to choose between self-sacrifice in carrying out their duty to treat and refusing to carry out their duty for the sake of their safety. Indeed, during the pandemic, some healthcare professionals experienced severe and frequent moral distress relating to patient care accompanied by high levels of intention to leave their position or profession (Sperling, 2021; Zulaihah et al., 2022), but many others were still willing to endorse altruistic self-sacrifice (Mamun et al., 2020). Expanding to the level of the organization, decision-makers in healthcare institutions were faced with the moral dilemma of mandating employees to get vaccinated as a condition of employment to ensure the safety of their patients, staff, and communities (Crist, 2022; Rachini, 2021) while prioritizing the individual’s right to autonomy and self-determination over medical treatment decisions (Olick et al., 2021). Healthcare professionals, including institutional decision-makers, may face a conflict between self-sacrifice and the well-being of the majority, or between the well-being of an in-group and the sacrifice of the minority. What makes them respond differently to this moral dilemma? Here, we investigated whether the context of moral conflicts (self-sacrifice vs. self-preservation) influenced the moral judgments of future healthcare professionals (i.e., healthcare students) and explored the individual traits modulating their judgments.

Using the trait-activation theory (TAT; Tett & Burnett, 2003), we propose to measure utilitarian tendencies on the Oxford Utilitarianism Scale (OUS), which has recently been developed, as a factor in individual differences for predicting utilitarian judgments in sacrificial moral dilemmas. The OUS provides a measure of trait-level individual differences in utilitarian tendencies resulting from two distinct utilitarianism aspects: a “negative” facet, called instrumental harm (IH), which reflects a permissive attitude toward harming others to achieve the greater good, and a “positive” facet, called impartial beneficence (IB), which reflects an unbiased concern for the well-being of all lives. Although previous studies of the antecedents of moral judgment and decision-making have mainly focused on the main effects of the IH and IB dimensions as a trait factor (Everett et al., 2018; Francis & McNabb, 2022; Navajas et al., 2021), the influence of the two components of utilitarian tendencies on utilitarian judgment may differ concerning the context. Therefore, it is imperative to examine how the IH and IB dimensions influence utilitarian judgments of healthcare-affiliated students when positioned as a member of the minority group, a member of the majority group, and in the third-person perspective in sacrificial moral dilemmas.

The present study makes the following contributions to the literature on moral dilemmas. First, it extends our knowledge by identifying conditions under which healthcare students’ utilitarian judgments may be related to their positioning as a member of a minority group, a member of a majority group, and in a third-person (neutral) perspective while making judgments in hypothetical moral dilemmas. Second, although personal traits have been proven to be antecedents linked to utilitarian judgment and decision-making (Choe & Min, 2011; Djeriouat & Trémolière, 2014; Gleichgerrcht & Young, 2013), little work has been done to explore the positive and negative aspects of trait-like utilitarian tendencies assessed through the OUS concerning the utilitarian judgments of healthcare students in terms of sacrificial moral dilemmas. Thus, this study may develop our understanding of how one’s utilitarian tendencies account for individual differences in utilitarian judgments.

## Theoretical Background and Hypotheses

### Self-sacrifice and utilitarian responses to moral dilemmas

In real-life moral dilemmas, people must decide whether to hold on to certain rights or obligations or to violate them in hope of a better outcome (Conway et al., 2018). Moral judgment studies have used sacrificial moral dilemmas incorporating a conflict in a hypothetical situation where the sacrifice of an innocent person must be endorsed to bring about the greater good. Responses to dilemmas can be used to identify people’s moral tendencies, which are a critical factor in moral judgment. A person who endorses sacrificial harm for the greater good is said to be making a utilitarian judgment, and a person who does not is said to be making a non-utilitarian judgment (or deontological judgment).

Moral judgments in dilemmas are significantly influenced by individual differences. For example, men have a greater tendency to make utilitarian judgments in moral dilemmas than women (Conway et al., 2018; Fumagalli et al., 2010), and older adults are more likely to make deontological judgments than younger adults (McNair et al., 2019). Moreover, concerning personal traits, diminished empathic concerns are positively associated with utilitarian judgments (Gleichgerrcht & Young, 2013), while habitual reappraisal as an emotion regulation strategy is negatively related to deontological judgment (Szekely & Miu, 2015).

However, there is another important factor that should be considered, namely, context. Earlier studies have demonstrated that context had a significant impact on moral judgment. For example, in moral dilemmas, the context of personal action (e.g., pushing an innocent person in front of a trolley) leads to deontological judgment more than the impersonal action context does (e.g., causing a trolley to switch tracks) as causing direct harm (i.e., personal action context) evokes a greater emotional response to moral violation than causing indirect harm (i.e., the impersonal action context) (Greene et al., 2001, 2004). Moreover, contexts, where participants can sacrifice an innocent person to save hundreds or thousands, cause them to make utilitarian judgments more intuitively than contexts where they can sacrifice an innocent person to save a few (Trémolière & Bonnefon, 2014). Beyond these structural manipulations in moral dilemmas, the personal values of the lives to be saved (e.g., an indeterminate adult versus the decider’s mother) can also have an impact on moral judgments, and objects with higher personal values have greater possibilities of being saved (Cohen & Ahn, 2016).

Because these various contextual factors influence people’s judgments, the self-involved context also significantly influences moral reasoning and judgments. Previous studies have found that participants endorse self-sacrifice to save several lives in moral dilemmas for reasons that may not be derived from social desirability but rather from self-protective tendencies and utilitarian reasoning (Mayer et al., 2021; Sachdeva et al., 2015). In other words, as the number of lives to be saved increases, people tend to make utilitarian judgments at their own expense, and this tendency falls as the number of lives to be saved shrinks to one or two, with the self-protective tendency becoming even greater (i.e., other-sacrifice) (Mayer et al., 2021). In a similar vein, in a moral dilemma, if the deciding individual is a member of the same group as the individuals at stake, the group’s benefit is more likely to be selected at the expense of an innocent person (Boccia et al., 2017; Lotto et al., 2014; Moore et al., 2008). These findings indicate that utilitarian reasoning is affected by self-protective tendencies (or self-interest), given the right context. In particular, if deciders have the space to rationalize their judgments, such as when they can justify them by the outcome of saving several lives, utilitarian judgments may be more prevalent.

Altruistic rationalizations support the explanation of such judgments. This means that interactions between groups are more competitive than those between individuals, as a decision-maker, being a member of a certain group, makes a self-oriented decision, rationalizing it as being for the sake of the in-group members (Pinter & Wildschut, 2012). This suggests that providing space for rationalization enables self-interest. If this principle is applied to the context of a moral dilemma, where a decision-maker is a member of the group at risk, the increased rate of utilitarian judgments may be derived from the rationalization of one’s selfish motives as if the judgment was made to save several people. We conjecture that a context that provides room for self-rationalization may induce self-interest. Accordingly, it is necessary to compare an other-involved context (i.e., classical moral dilemmas) with the two categories of self-involved context to explore the distinct effects of these contexts on healthcare students’ moral judgments.

In our exploratory study, we focus on the effects of perspective switching and utilitarian thinking on healthcare students’ moral judgments and compare them with those of non-healthcare students to better understand the moral characteristics of healthcare students. To the best of our knowledge, no previous studies have been conducted to explore how moral reasoning, context, and educational background independently and jointly influence moral judgments. For this reason, we conduct a detailed study to identify healthcare students’ moral reasoning. To this end, we tested their moral judgments in serial dilemma scenarios, manipulating participants’ perspectives (the *neutral* condition of an observer, the *self-in-minority* condition as a minority person or group, and the *self-in-majority* condition as a member of the majority group). Through this manipulation, we sought to remove the influence of individual differences and to check the effects of self-protective tendency and altruistic rationalization on moral judgments depending on the context.

As noted above, in general, people tend to accept their self-sacrifice for others’ well-being in dilemma scenarios, but they also have a self-protective tendency (Volz et al., 2017). The self-protective tendency may increase under the *self-in-majority* condition, where there is room to rationalize their selfish motivation as if one’s judgment is for the sake of the in-group members, but there is no room in the other conditions.

Therefore, we propose the hypothesis that utilitarian responses are the most dominant in the *self-in-majority* condition, and there is no significant difference between the responses in the *neutral* and *self-in-minority* conditions.

### Utilitarian thinking and moral judgments

Taking utilitarianism as an appropriate framework for the evaluation of moral judgment (Bartels & Pizarro, 2011), numerous studies have examined the factors affecting utilitarian judgments in sacrificial moral dilemmas. One of the key factors is individual disposition because moral judgment and actions require ethical competence influenced by dispositional traits (Pohling et al., 2016). Several studies have investigated the influence of personality traits on utilitarian moral judgment. For example, antisocial personality trait constructs, such as psychopathy, Machiavellianism, and life meaninglessness, are positively related to the endorsement of utilitarian solutions (Bartels & Pizarro, 2011). In a study of emotion and moral judgment, Choe and Min (2011) found that the trait anger was positively correlated with utilitarian judgment, but trait disgust and trait empathy were negatively correlated. A more recent study has demonstrated that the politeness aspects (e.g., respectfulness and etiquette) of agreeableness are positively associated with deontological judgments, whereas the intellectual aspects of openness (e.g., curiosity and cognitive engagement) are positively associated with utilitarian judgments (Smillie et al., 2021).

In 2018, Kahane and colleagues proposed a new framework for understanding utilitarian psychology, conceptualizing two aspects of utilitarianism: (a) instrumental harm (IH), which captures the extent to which one is willing to accept harming and even killing others to achieve the greater good, and (b) impartial beneficence (IB), which reflects the willingness to endorse the promotion of everyone’s welfare without regard to the physical, emotional, or relational distance between the actor and the beneficiary. To evaluate an individual’s position in both the positive (IB) and negative (IH) components of utilitarianism, Kahane et al. (2018) developed and validated a novel measure – namely, the Oxford Utilitarianism Scale (OUS). The authors compared the OUS with measures of individual difference thought to relate to utilitarianism and demonstrated the scale’s convergent, discriminant, and predictive validity. Specifically, individuals with greater levels of empathic concern, identification with the whole of humanity, and concern for future generations were found to exhibit greater IB but lower IH. In addition, individuals with higher levels of psychopathic traits exhibited greater acceptance of causing harm for the greater good, but those with higher levels of religiosity exhibited a greater endorsement of the impartial maximization of the greater good. Furthermore, participants who scored higher on the IH and IB subscales were more likely to endorse the utilitarian option in sacrificial moral dilemmas. These results could be interpreted to suggest that the two core aspects of utilitarian tendencies, measured using the instrumental harm subscale (OUS-IH) and impartial beneficence subscale (OUS-IB), are related but distinctive constructs, and play different roles in affecting moral judgment and decision-making.

We adopted the trait-activation theory (TAT; Tett & Burnett, 2003) to determine the relevance of sacrificial moral dilemmas for the expression of OUS-IH and OUS-IB. The TAT emphasizes the person-situation interplay, suggesting that people express their traits when presented with trait-relevant situational cues. According to the TAT, sacrificial moral dilemmas in hypothetical situations that make two aspects of trait-level utilitarian tendencies, namely, IH and IB, relevant may afford healthcare and non-healthcare students opportunities for their IH and IB to be activated and expressed, thereby influencing their endorsement of utilitarian solutions. For these reasons, healthcare students who have a greater willingness to harm (IH) or help (IB) for the greater good are expected to advocate a more utilitarian approach to moral judgments in sacrificial dilemmas. Thus, we expect the OUS-IH and OUS-IB to be significantly associated with the endorsement of utilitarian judgments made with different perspectives in sacrificial dilemmas in a sample of healthcare and non-healthcare students. However, it is relatively unknown how far each tendency is activated concerning the major fields of study that participants choose at college. As such, the present study employed an exploratory approach to further our understanding of how utilitarian tendencies can be characterized as individual difference factors by examining whether the relationship between each tendency and sacrificial moral judgment varies depending on a college major.

## Method

### Participants and procedure

The participants in this study were 339 Korean undergraduates who contributed in exchange for course credit. The sample included 227 healthcare students enrolled in medicine (*N* = 121; 66 men; *M*_age_ = 24.97, *SD* = 2.38) and dentistry (*N* = 106; 60 men; *M*_age_ = 20.89, *SD* = 1.01), along with 112 non-healthcare students (35 men; *M*_age_ = 22.84, *SD* = 2.23). After providing informed consent, all the respondents were invited via e-mail to take a web-based survey, including measures of empathy, emotion regulation skill, utilitarian thinking, and moral judgment. Finally, demographic information was collected.

### Measures

**Oxford Utilitarianism Scale** (**OUS**) (see Table 1 in the Appendix)*.* The OUS (Kahane et al., 2018) includes nine items: five drawn from the impartial beneficence subscale (OUS-IB) and four from the instrumental harm subscale (OUS-IH). The two components were rated on a 7-point-Likert scale ranging from 1 (strongly disagree) to 7 (strongly agree), with Cronbach’s α value of.60 for OUS-IB and.75 for OUS-IH. Higher scores on the OUS indicate a greater tendency toward the corresponding component of utilitarian thinking.

**Table 1:** Correlations among all variables by college major

Measure	1	2	3	4	5	6
1. EC	-					
2. CR	**.34(.38)**	-				
3. OUS-IB	**.28**(.24)	.07(**.30**)	-			
4. OUS-IH	-.14(.10)	.01(.17)	**.24(.48)**	-		
5. Neutral	-.13(-.10)	.08(.02)	.01(-.17)	**.41**(.20)	-	
6. Self-in-minority	.06(.10)	**.22(.21)**	.17(.24)	**.19**(.19)	**.50**(**.46**)	-
7. Self-in-majority	-.15(.00)	.04(-.10)	-.07(**-.32**)	**.38**(.00)	**.75**(**.69**)	**.38**(.17)

*N* = 227 (112). Numbers inside parentheses refer to correlation coefficients in the non-healthcare group. *EC* Empathic Concern; *CR* Cognitive Reappraisal; *OUS-IB* Oxford Utilitarianism Scale-Impartial Beneficence; *OUS-IH* Oxford Utilitarianism Scale-Instrumental Harm; two-tailed *p*-values are indicated by text formatting as: < .05 and < **.01**

**Moral Dilemmas** (see Table 2 in the Appendix). An adaptation of Moore et al. (2008) moral judgment task was employed to evaluate the extent to which the participants agreed with the utilitarian act (e.g., save the majority at the expense of the minority) taken by a central person in a moral dilemma scenario from among the Crying Baby, Burning Building, and Submarine scenarios (see Supplementary Materials S1). These scenarios were used in place of the medical context scenarios that the medical students might be more used to so that all participants would encounter a similarly unfamiliar judgment to ensure that their moral judgments were not influenced by their educational background. The Crying Baby scenario, for example, represents a hypothetical dilemma in which a parent has no choice but to smother his or her crying baby to save others hiding from murderous enemy soldiers, and the participants were asked to rate their endorsement of this action on a scale of 1 (strongly disagree) to 6 (strongly agree). The other two dilemmas likewise involve killing one person to save a greater number of people, pitting the desire to maximize good consequences against causing harm. After evaluating all three scenarios from the perspective of a *neutral* third party (such as an observer), all participants were asked to review the same scenarios again using the same scale. Half of the participants were then asked to assess the utilitarian action in each scenario (e.g., smothering the baby to save other civilians in the Crying Baby scenario) from the perspective of a member of the minority group in the scenario (i.e., *self-in-minority*; the parent’s^1^ role in the Crying Baby scenario), followed by doing so from the perspective of a member of the majority group in the scenario (i.e., *self-in-majority*; the role of the other townspeople in the Crying Baby scenario). The other half were asked to make the same assessment of the utilitarian action in the opposite order, first in-majority and then in-minority contexts.

**Table 2:** Moderating analysis of utilitarian thinking on moral judgments in the *self-in-majority* context

Variables	Moral judgements		
	Model 1	Model 2	Model 3
Step 1: Control variables			
Age	.02	.03	.05
Gender	.11*	.13**	.14**
EC	-.14*	-.04	-.04
CR	.05	.02	.03
Step 2: Main effects			
OUS-IH		.36***	.21*
OUS-IB		-.26***	-.41***
Major		-.06	-.06
Step 3: Interaction			
Major x OUS-IH			.20*
Major x OUS-IB			.19*
*R*^*2*^	.03	.16	.19
*ΔR*^*2*^		.14	.03
*ΔF*		18.00***	6.72**

Standardized coefficients were represented; *EC* Empathic Concern; *CR* Cognitive Reappraisal; *OUS-IB* Oxford Utilitarianism Scale-Impartial Beneficence; *OUS-IH* Oxford Utilitarianism Scale-Instrumental Harm; * *p* < .05**;** ** *p* < .01; *** *p* < .001

**Control Variables** (see Table 3 in Appendix). We measured empathic concern (EC) and cognitive reappraisal (CR) as control variables as these factors are personal traits associated with moral judgments, altruism, and helping behavior (Batson et al., 1988; Gleichgerrcht & Young, 2013; Lebowitz & Dovidio, 2015; Szekely & Miu, 2015). EC is an affective aspect of empathy, and it was assessed using the seven-item EC component of the Interpersonal Reactivity Index created by Davis (1983). The component was rated on a 5-point Likert scale, ranging from 0 (does not describe me well) to 4 (describes me very well), with a Cronbach’s α value of.72. Higher scores on the EC indicate greater levels of concern and sympathy for others. We adopted six items from the Emotion Regulation Questionnaire developed by Gross and John (2003) to evaluate an individual’s habitual use of CR as an emotion regulation strategy to downregulate negative emotions by reinterpreting emotional situations. This component was rated on a 7-point Likert scale, ranging from 1 (strongly disagree) to 7 (strongly agree), with a Cronbach’s α value of.84. Higher scores on the CR indicate greater use of the emotional regulation strategy.

## Results

### Group differences in responses to the dilemmas

To determine the differences between major groups in response to moral dilemmas, a repeated measures analysis of covariance, controlling for EC and CR, was conducted, with ratings of the utilitarian action as the dependent measure and major (healthcare vs. non-healthcare) and context (neutral vs. self-in-minority vs. self-in-majority) as the independent variables. The test results identified a significant main effect of context, *F*(2, 670) = 13.96, *p* < 0.001, *η*_*p*_^*2*^ = 0.04, indicating that participants’ agreement ratings for the utilitarian action were affected by how they viewed themselves, namely, whether as a minority group member, a majority group member, or a third party. The main effect for college major was not observed, *F*(1, 335) = 0.99, *p* = 0.32, *η*_*p*_^*2*^ = 0.003, but there was a significant major x context interaction, *F*(2, 670) = 3.49, *p* = 0.04, *η*_*p*_^*2*^ = 0.01, indicating that the impact of self-perception in the context of minority, majority, and third party groups on the ratings for the utilitarian action differed between college majors.

**Fig. 1:**
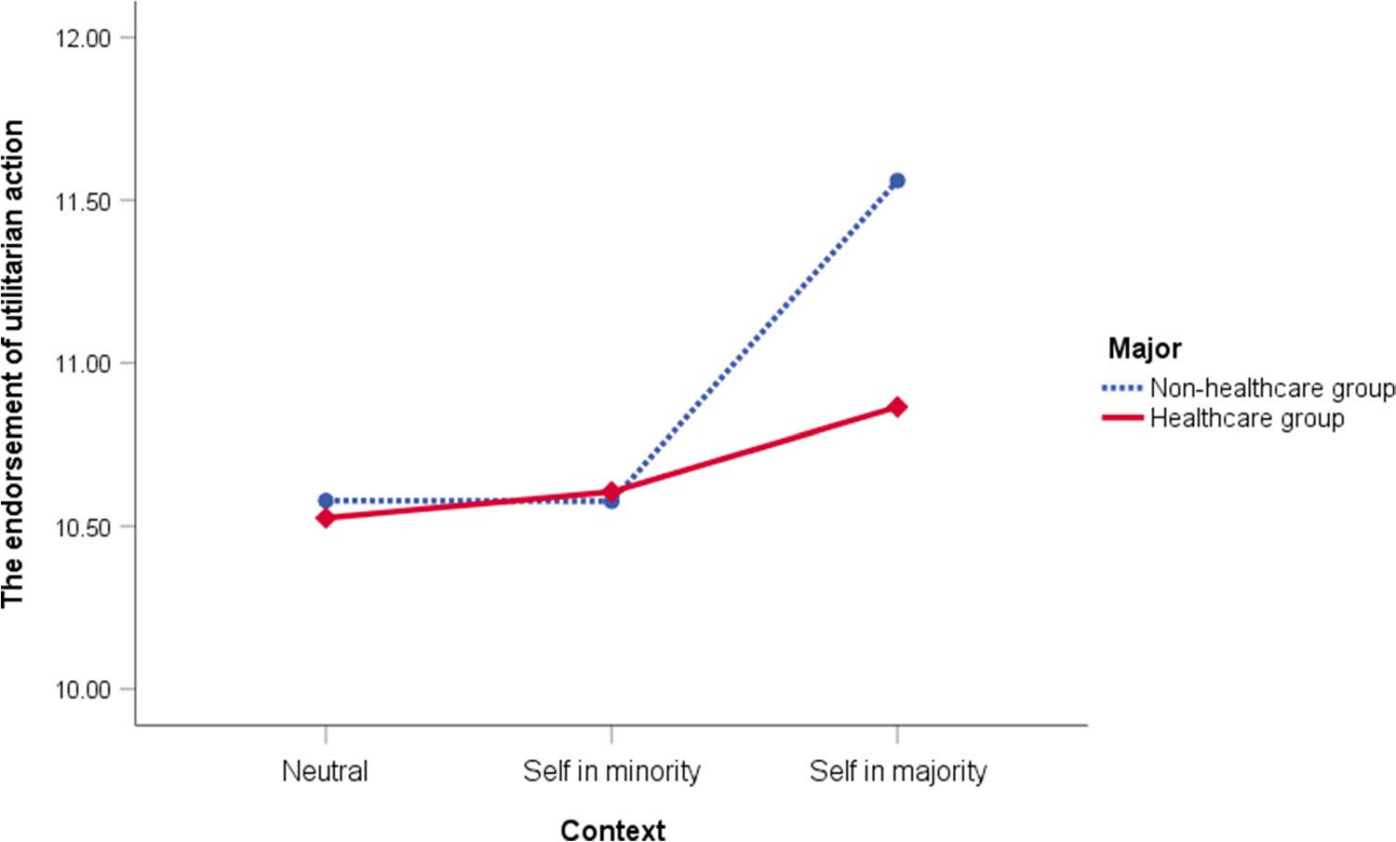
The endorsement of utilitarian action of the three dilemma contexts between the two major groups

Subsequent analyses were conducted using Bonferroni multiple comparisons test to identify possible interaction effects. Although both major groups gave significantly higher agreement ratings for the utilitarian action in the *self-in-majority* context than in the *neutral* context, *t*(337) = 2.69, *p* = 0.02., Cohen’s *d* = 0.13 for the healthcare group and *t*(337) = 5.46, *p* < 0.001, *d* = 0.42 for the non-healthcare group, the participants in the healthcare group were less ready to endorse the action than their non-healthcare counterparts in the *self-in-majority* context, *t*(337) = 2.29, *p* = 0.02, *d* = 0.27 (Fig. 1).

However, no significant differences were observed in the agreement ratings between the *self-in-minority* and *neutral* contexts, *t*(337) = 0.05, *p* = 1.00., *d* = 0.02 for the healthcare group and *t*(337) = 0.00, *p* = 1.00., *d* = 0.00 for the non-healthcare group, such that the gap that favored the non-healthcare group disappeared in both the *self-in-minority*, *t*(337) = 0.18, *p* = 0.86, *d* = 0.02, and the *neutral*, *t*(337) = 0.49, *p* = 0.62, *d* = 0.06, contexts.

### Two-dimensional impacts of utilitarian thinking on moral dilemma judgments

Table 1 presents the correlations between the study variables across college major groups.

Bivariate correlations were performed to determine whether the two aspects of utilitarian tendencies—that is, IH and IB—were associated with the agreement ratings for each group for the sacrificial action in moral dilemmas across the given contexts. In the neutrally framed sacrificial dilemmas (i.e., in the *neutral* context), the agreement ratings for the utilitarian actions significantly correlated with the OUS-IH, *r* = 0.41, *p* < 0.01 but not with the OUS-IB, *r* = 0.01, *p* = 0.89, in the healthcare group. A test of significance was conducted between the two correlations (Steiger, 1980), and it was shown that they significantly differed, *z* = 6.24, *p* < 0.01. This pattern was also identified in the non-healthcare group, which implied that the correlation of utilitarian judgments in neutrally framed dilemmas with the OUS-IH, *r* = 0.20, *p* = 0.04, was higher than the correlation with the OUS-IB, *r* = -0.17, *p* = 0.08, with a significant difference between these two correlations, *z* = 3.71, *p* < 0.01. These findings are consistent with previous research work (Navajas et al., 2021), which suggests that sacrificial dilemmas restrict the attention to the negative aspects of utilitarianism, namely, the permissibility of causing IH.

By contrast, the agreement ratings for the utilitarian action in sacrificial dilemmas in the *self-in-minority* variant were correlated significantly with the OUS-IB, *r* = 0.17, *p* = 0.01, as well as with the OUS-IH, *r* = 0.19, *p* < 0.01, in the healthcare group, indicating that the two correlations were non-significantly different, *z* = 0.27, *p* = 0.39. The same pattern was observed in the non-healthcare group, indicating that utilitarian judgments in sacrificial dilemmas in the *self-in-minority* variant were significantly correlated with the OUS-IH, *r* = 0.19, *p* = 0.046, as well as with the OUS-IB, *r* = 0.24, *p* = 0.01, exhibiting a non-significant difference between the two correlations, *z* = 0.54, *p* = 0.30.

Finally, in the healthcare group, the agreement ratings for the utilitarian action in sacrificial dilemmas in the *in-majority* variant were significantly correlated with the OUS-IH, *r* = 0.38, *p* < 0.001, but not with the OUS-IB, *r* =  − 0.08, *p* = 0.24. However, the opposite pattern was observed for the non-healthcare group, whose agreement ratings were significantly correlated with the OUS-IB, *r* =  − 0.32, *p* < 0.001, but not with the OUS-IH, *r* = 0.002, *p* = 0.99. These correlational data lead us to conduct moderated regression analysis to examine whether the degree to which the two dimensions of utilitarianism exert a differential impact on the endorsement of utilitarian solutions varies as a function of college major, expecting to account for the observed group differences in the *self-in-majority* context. Controls^2^ (age, gender [dummy-coded; 0 = male; 1 = female], EC, and CR) were included in the first step, followed by all predictors— major (dummy-coded; 0 = non-healthcare; 1 = healthcare), OUS-IB and OUS-IH (mean-centered)—in the second step. In the third step, we entered two interaction terms: major x OUS-IH and major x OUS-IB. As presented in Table 2 (Model 1), the results revealed a significant effect for gender, *β* = 0.11, *p* = 0.04, indicating that females were more likely than males to endorse the utilitarian action in the *self-in-majority* context. The same pattern also applied to EC, *β* = *-*0.14, *p* = 0.02, indicating that participants with higher empathic concern revealed greater endorsement of utilitarian action. As expected, a significant main effect was detected for both the OUS-IH, *β* = 0.36, *p* < 0.001, and the OUS-IB, *β* = *-*0.26, *p* < 0.001 (Model 2), suggesting that the two core aspects of utilitarian tendencies were significantly related to the endorsement of utilitarian solutions in the *self-in-majority* context. After controlling for the main effects, the major x OUS-IH interaction (Model 3) remained significant for predicting the endorsement of utilitarian solutions, *β* = 0.20, *p* = 0.04. This pattern was found reliable for the major x OUS-IB interaction (Model 3), *β* = 0.19, *p* = 0.049.

To better understand the precise nature of the above interactions—in this case, major x OUS-IH and major x OUS-IB, a simple slopes analysis and the Johnson-Neyman procedure (Johnson & Fay, 1950) were conducted using R software (R project version 4.1.1).

**Fig. 2:**
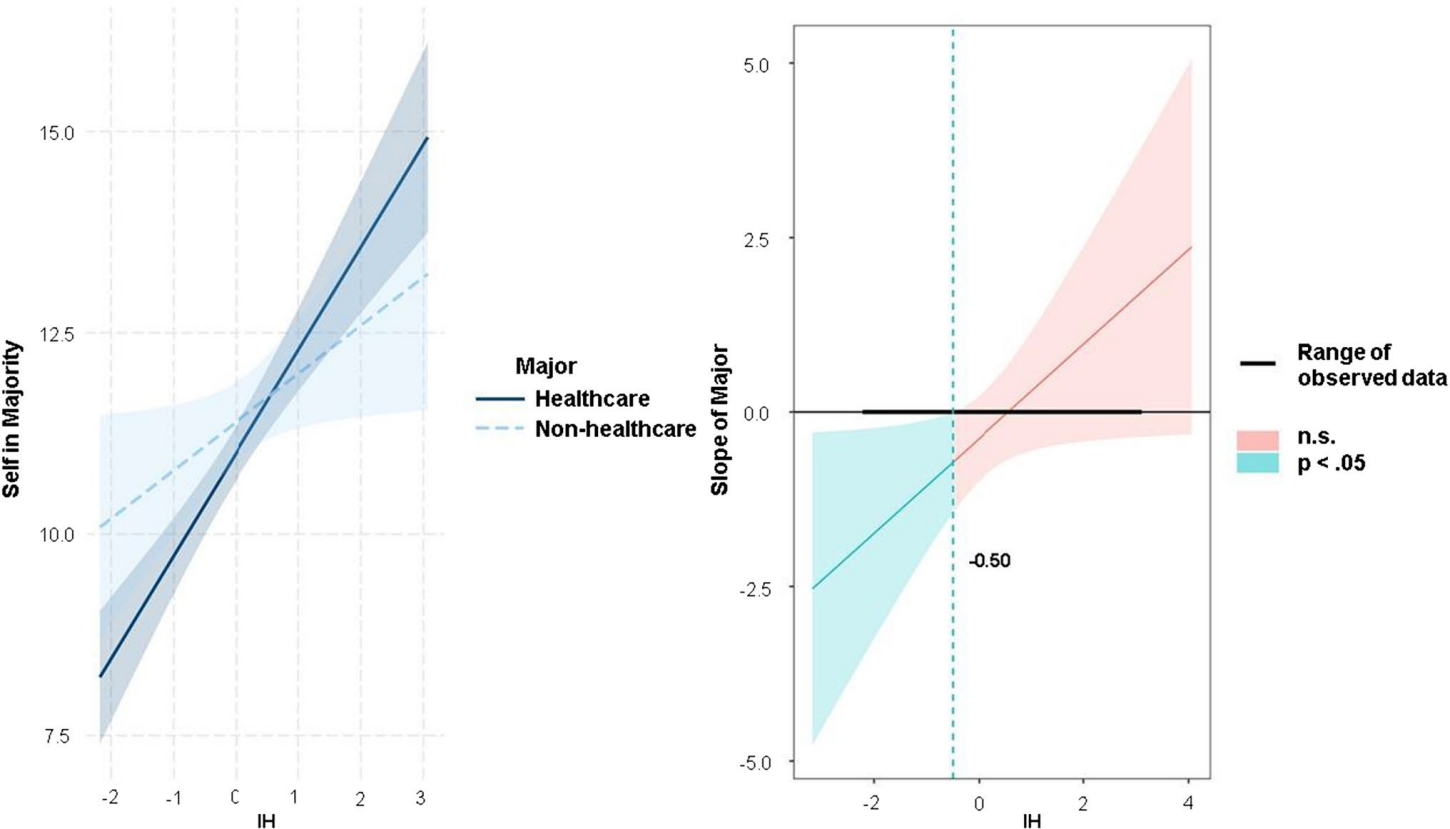
Interaction effect of the OUS-IH and College Major on Utilitarian Endorsement (left) and corresponding Johnson-Neyman plot (right)

As presented in Fig. 2 (left side), the slope of the regression line of IH concerning the endorsement of a utilitarian solution was steeper for the healthcare than the non-healthcare group. The results of the Johnson-Neyman technique showed that at low levels of the OUS-IH (standardized z scores of -0.50 and lower; Fig. 2, right side), IH affected the healthcare group’s agreement ratings for utilitarian action to a greater extent than it affected the ratings provided by the non-healthcare group. This finding, coupled with the fact that the healthcare group (*M* = 3.10, *SD* = 0.99) had a significantly lower score on the OUS-IH than the non-healthcare group (*M* = 3.34, *SD* = 0.98), *t*(337) = 2.15, *p* = 0.03, *d* = 0.27, can be interpreted to suggest that even when placed in the *self-in-majority* context where the endorsement of utilitarian outcomes can more easily be rationalized, the healthcare group was more reluctant than the non-healthcare group to endorse sacrificing the minority for the sake of the majority because their stronger tendency to devalue the acceptance of harm for the greater good was activated and then expressed in their moral judgment.

**Fig. 3:**
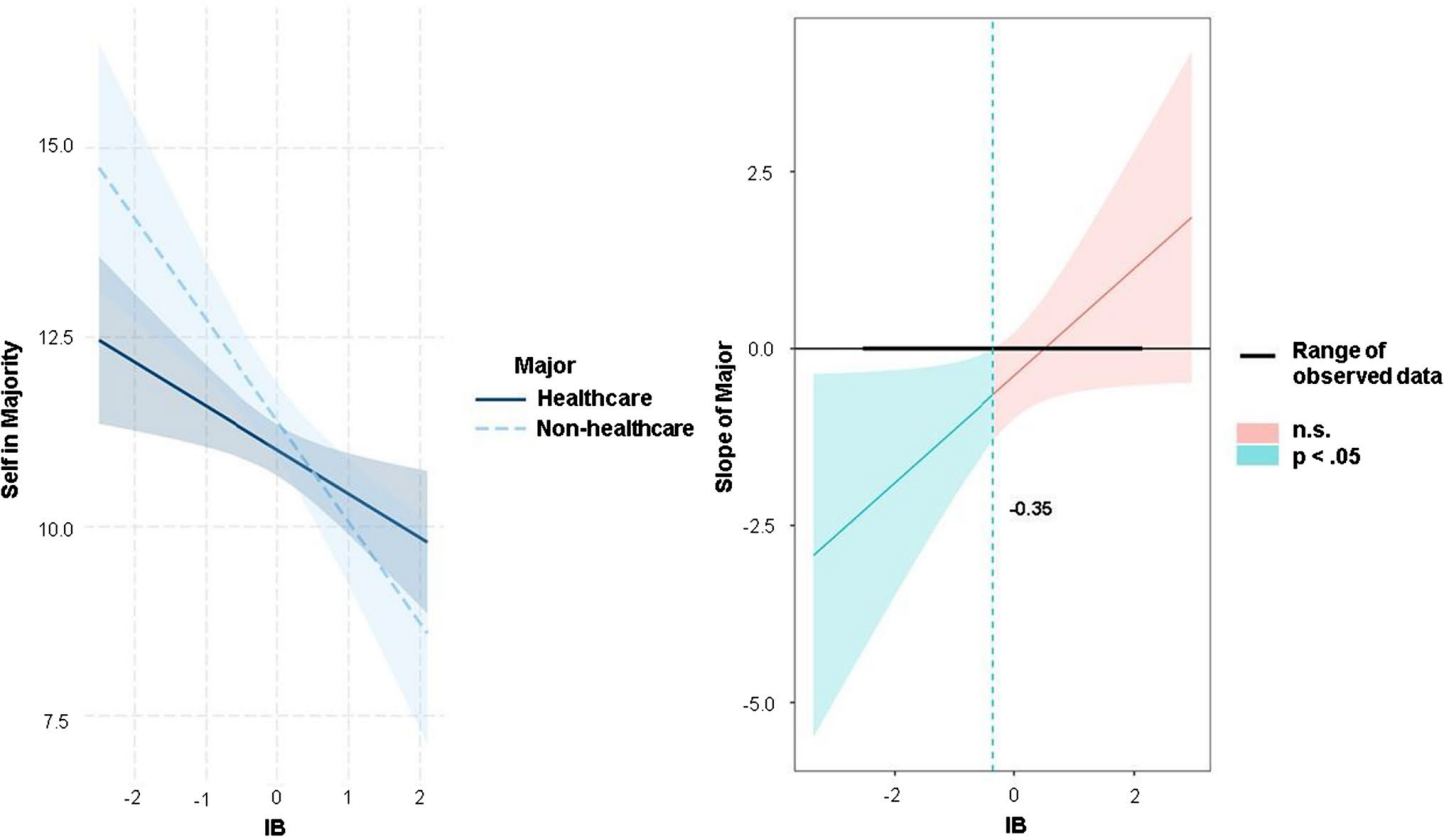
Interaction effect of the OUS-IB and College Major on Utilitarian Endorsement (left) and corresponding Johnson-Neyman plot (right)

Conversely, Fig. 3 (left side) presents a steeper slope of the regression line of IB on the endorsement of utilitarian solutions for the non-healthcare than for the healthcare group. Using the Johnson-Neyman test, IB affected the non-healthcare group’s agreement ratings for utilitarian action to a greater extent than it affected the ratings provided by the healthcare group at low levels of the OUS-IB (standardized z scores of -0.35 and lower; Fig. 3, right side).

## Discussion

This study investigated the impact of context (neutral vs. self-in-minority vs. self-in-majority) and its interplay with college majors (healthcare vs. non-healthcare) on utilitarian judgments in sacrificial moral dilemmas. Our results indicated that both major groups expressed a stronger pro-utilitarian position in the context of judgments in moral dilemmas where the *self-in-majority* context was present than in the two other contexts, with healthcare students being more reluctant to endorse the utilitarian action than their non-healthcare counterparts in the *self-in-majority* context.

Utilitarian endorsements were the most salient in the *self-in-majority* context, regardless of college major, which can be interpreted using the altruistic rationalization hypothesis: individuals can rationalize choices that benefit themselves when deciding in groups, arguing that the other members of the group will also benefit from it (Insko et al., 1987). According to this explanation, the participants in the *self-in-majority* context may have rationalized their agreement ratings for taking utilitarian action as flowing from a concern for the benefit of the majority, although the underlying reason behind their endorsements may be to save their own lives. This concern for the majority, put another way, may be morally hypocritical and self-interested rather than sincere. Conversely, it is impossible in both the *neutral* and *self-in-minority* contexts for participants to have the opportunity for altruistic rationalization as they do not themselves benefit from endorsing the utilitarian action in each context. For this reason, we conjecture that the participants in this study, regardless of their major, are more likely to express a stronger pro-utilitarian position when making moral dilemma judgments in the presence of opportunity for altruistic rationalization. This argument is supported by two studies (Pinter & Wildschut, 2012; Pinter et al., 2007) that investigated the role of altruistic rationalization in the context of the prisoner’s dilemma game (PDG) by comparing the PDG decisions of participants who made choices that either partially or fully determined the earnings of in-group members within a three-person set (and who could thus rationalize their competitive choice as being enacted for the sake of the two other members) together with participants whose choices did not affect the earnings of in-group members within a three-person set (and who thus had no opportunity for altruistic rationalization). The results indicated that the proportion of competitive PDG choices was greater when opportunities for altruistic rationalization were present; however, this effect was only significant for participants who scored low in terms of dispositional proneness to guilt. Drawing on the findings of Pinter and colleagues (2007) and Pinter and Wildschut (2012), we can interpret our results to mean that individuals are more likely to make self-benefiting decisions that masquerade as benefiting in-group members when they have opportunities for altruistic rationalization, resulting in more salient utilitarian endorsements in the *self-in-majority* context.

Next, the finding that the differences between the healthcare and non-healthcare groups emerge only in the *self-in-majority* context furthers our understanding of conditions under which healthcare students’ inclination to engage in self-sacrificial behavior becomes salient. Even with the possibility that the endorsement of utilitarian actions could be taken for self-interest (i.e., saving their own lives) as flowing from in-group beneficence (i.e., saving a great number), healthcare students in the *self-in-majority* context were found to be more reluctant to endorse utilitarian action than their non-healthcare counterparts. Notably, we shed new light on the reasons for which these group differences emerged only in the *self-in-majority* context. It was found that the influences exerted on utilitarian judgments by the two dimensions of utilitarian thinking varied as a function of college majors. When considered together with IH and IB on the OUS, the instrumental harm component (OUS-IH) emerged as a significant predictor of utilitarian judgments in the healthcare group, whereas the impartial beneficence component (OUS-IB) significantly predicted utilitarian judgments in the non-healthcare group.

A possible explanation indicating why the IH component was more influential in forming healthcare students’ utilitarian judgments in the *self-in-majority* context relates to their professional identity—a representation of self, that is, formed by the internalization of the characteristics, values, and norms of the medical profession (Holden et al., 2012) throughout a long process of interaction with the medical curriculum (Monrouxe, 2010), resulting in altered ways of thinking, behaving, and feeling to resemble those of a doctor to a greater degree (Cruess et al., 2014). For example, healthcare students are encouraged to develop their professional identity by taking an oath of ethical commitment or participating in white coat ceremonies in a curricular event to enhance their humanism and professionalism at the time of their admission to and graduation from medical school (Karnieli-Miller et al. 2013). In the healthcare sector, where healthcare professionals are expected to provide their patients with the best possible care available (Bitter et al., 2013), in a way consistent with a deontological ethic that is patient-centered, highly valuing the particularity of each patient (Garbutt & Davies, 2011; Wesarat & Mathew, 2021), healthcare students’ moral tendencies can be socially reinforced to favor the deontological basis of medical care, emphasizing that each patient is an end in themselves but not a means to be used to attain anything else (Heath, 2004). This interpretation is supported by our data that healthcare students had significantly lower scores than their non-healthcare counterparts on the OUS-IH, indicating that healthcare students are less disposed to instrumentally using, severely harming, or even killing innocent people to promote the greater good than non-healthcare students. We draw on the TAT (Tett et al., 2013) to explain how healthcare students can activate and express lower trait-like IH in the form of moral judgments when placed in the *self-in-majority* context. According to the TAT, an individual’s trait is activated if he or she is exposed to situations where cues that are relevant to the arousal of that trait are provided. That is, the *self-in-majority* context acts as a strong situational cue that can provide healthcare students with the opportunity to express their lower trait-level tendency to allow harm to occur for the greater good, causing them to be more reluctant to endorse the sacrifice of the minority for the sake of the majority. This may result in marked group differences in utilitarian judgments in the *self-in-majority* context.

Although it was not the main question of this study, it is noteworthy that women endorsed utilitarian solutions to a significantly greater degree than men in the *self-in-majority* context. These results differ from previous results showing that men are more likely to make utilitarian judgments than women in conventional moral dilemma scenarios (Conway et al., 2018; Fumagalli et al., 2010). We suggest two possibilities for our results. First, it may be that the gender difference in risk-taking has influenced them. Previous studies show that men were more likely to make judgments about self-sacrifice or help strangers than women (Lyons, 2005; Swann et al., 2014). These findings reflect that women are more averse to risk-taking prosocial judgments or behavior than men, due to their stronger risk aversion (Croson & Gneezy, 2009; Diekman & Clark, 2015). Therefore, women may have made more utilitarian judgments in the *self-in-majority* context because they did not want to take the risk of self-sacrifice. The other possibility is in-group favoritism. The gender difference in prosocial behavior depends on its type. As mentioned above, prosocial behavior that requires risk-taking is more performed by men than women, and caring for others (especially the socially disadvantaged) or self-sacrifice for people in close relationships tends to be more dominant in women than men (Diekman & Clark, 2015). Accordingly, in the *self-in-majority* context, as women perceived their group members as individuals in a close relationship, they may have made more utilitarian judgments than men to protect the group members. Although this study suggests these hypothetical explanations due to the limitations of the study design, a detailed analysis should be performed in future studies to determine whether the gender difference continues to be replicated even in diversified contexts, such as the medical context of COVID-19, and what is the cause of the differences.

### Theoretical implications

This has theoretical implications for the literature on utilitarianism using sacrificial moral dilemmas. First, it identifies the condition under which people are likely to endorse utilitarian solutions to sacrificial dilemmas. In addition, it demonstrates that individuals’ endorsement of utilitarian solutions to sacrificial dilemmas can be contextualized concerning the perspective taken in the dilemma contexts, at least in the samples of healthcare and non-healthcare students. Specifically, in our study, regardless of their college major, the participants give higher agreement ratings for the utilitarian action in sacrificial dilemmas in the *self-in-majority* variant than in either the *neutral* or *self-in-minority* variants. The findings can be explained by the altruistic rationalization hypothesis (Pinter et al., 2007), which suggests that when placed in the majority group and when therefore allowed altruistic rationalization, the participants are more likely to become utilitarian, rationalizing their endorsement of utilitarian solutions, flowing from a concern for benefiting the majority, although the reason for their utilitarian judgment may have more relevance to their intention to save their own lives. Thus, the presence of an opportunity to rationalize self-benefiting choices and behavior may be a situational cue that facilitates both the healthcare and non-healthcare groups to become more utilitarian in their outlook. More importantly, the healthcare group appeared less vulnerable to this situational influence, indicating that they were more reluctant than their non-healthcare counterparts to accept harm to others for the greater good in sacrificial dilemmas in the *self-in-majority* variant.

Second, this study contributes to the literature by demonstrating that the IH component serves as a critical dispositional factor that can explain why the healthcare group is less utilitarian (more deontologically biased) in their moral judgments in the *self-in-majority* context than the non-healthcare group. Furthermore, IB played a key role in explaining individual differences in utilitarian judgments of healthcare and non-healthcare students in the *self-in-minority* context. Its examination of the impact of endorsing IH and IB on utilitarian judgment, particularly in its comparison of a sample of healthcare and non-healthcare students, makes the present study an enriching addition to the moral judgment literature.

### Practical implications

From a practical viewpoint, our findings indicate an educational approach for healthcare students. In our study, healthcare students tended to ponder the minimization of harmful outcomes and human rights violations rather than consider IB in moral dilemmas. Their moral reasoning could be derived from the deontological basis of medical education and clinical practice, which promotes a concern for the patient first. However, medical professionals may sometimes be required to think flexibly in response to novel or changing circumstances. During a pandemic, because resources are finite, medical professionals must find the most cost-effective way to secure overall well-being (i.e., utilitarian thinking). From a public health viewpoint, it is also clear that health professions should seek to promote health equity at the community level (Browne et al., 2012). Therefore, educational interventions that allow individuals to reflect on dilemma situations and share their opinions with colleagues, such as problem-based learning classes using various real cases, will be required, which can improve their coping strategies for the situation and professional development (Khatiban et al., 2019; Kurtz & Starbird, 2016; Ribeiro et al., 2021).

### Limitations and future research

The present study identified differences concerning moral judgments and personal attributes between the two major groups, although this was done in the context of some limitations. First, the participants’ responses to moral dilemma scenarios were measured to investigate the differences in students’ moral reasoning between the healthcare and non-healthcare groups. Although responses to the scenarios predicted personality traits or behaviors (Dickinson & Masclet, 2019; Koenigs et al., 2012), our findings have limited ability to predict group differences in actual moral behavior, as our task did not measure actual behavior. In future research, other experimental methods, such as virtual reality, are required to identify the group differences in actual behaviors as well as moral thinking.

Second, our moral dilemma scenarios may merely measure the respondents’ judgment. In other words, responses to the scenarios include various motives, such as altruistic or egoistic ones; thus, it is unclear what motives the responses came from. To identify whether differences in the areas of scholastic focus affect motives for moral judgment, it is necessary to measure additional scales for various motives or introduce a new experimental paradigm that can differentiate between antisocial and prosocial motivation in utilitarian judgments, such as a process-dissociation approach (Conway et al., 2018).

Third, our moral dilemma scenarios had no relationship to practical medical contexts. Because contexts can impact the respondents’ judgments (Hester & Gray, 2020), if both healthcare- and non-healthcare-affiliated students are immersed in the role of healthcare professionals in the medical context, this role can have an impact on their judgments. For example, in other contexts, the identities of healthcare-affiliated students may be intensified, leading them to increasingly make self-sacrificial judgments, or the altruistic rationalization of non-healthcare-affiliated students may be diminished due to the alteration of their identity. Hence, it is necessary to generalize our results by conducting additional studies with scenarios in the medical context.

Fourth, the findings observed in healthcare students may not be generalizable to healthcare professionals. Although a few studies found the two groups similar in their knowledge of disaster medicine (Su et al., 2013), attitudes toward persons with physical disabilities (Satchidanand et al., 2012), and ability to distinguish between true and false coronavirus-related news stories (Grüner & Krüger, 2021), future studies should be designed to include mixed samples of healthcare professionals (e.g., doctors and nurses) and students to extend the generality of our findings by comparing their moral judgments made adopting multiple perspectives.

Finally, cultural contexts may also affect moral judgments and values, in that national culture plays an influential part in shaping individuals’ morality (Graham et al., 2016). As South Korea is characterized by a greater tendency toward collectivist culture, in which individuals are expected to align themselves with group norms and prioritize group over individual decisions (Hofstede, 1984; Triandis, 1990), endorsement of apparently utilitarian solutions to sacrificial dilemmas in the *self-in-majority* context can be more likely to be perceived as socially acceptable. Therefore, it is worthwhile exploring whether the present study results observed from the South Korean sample are indeed generalizable to healthcare student samples in an individualist culture where autonomy, individual initiative, and the primacy of personal goals over in-group goals are emphasized (Hofstede, 1984; Triandis, 1990), which would help enhance the external validity of our findings.

## Supplementary Information

Below is the link to the electronic supplementary material.

Supplementary file1 (DOCX 26 KB)

## Data Availability

The datasets generated during the current study are available from the corresponding author on reasonable request (https://osf.io/t83ax/).

## Footnotes

1 Although the parent may be part of the majority group in terms of benefiting from killing the baby (i.e., protecting their lives), we considered the parent with their baby as a minority group because unlike other civilians, the parent can lose more from sacrificing their baby than they can gain from it.

2 As the *self-in-majority* context provides the opportunity to rationalize one’s own selfish motivation, individuals confronted with the majority group perspective on the given dilemma are expected to respond quite differently to the opportunity, depending on their demographic characteristics—in this case, their age and gender (Rhim et al., 2020; Swann et al., 2014). To control for the effect of individual differences on the endorsement of utilitarian action in the *self-in-majority* context, age and gender were included as further covariates in the moderated regression analysis, along with EC and CR.
