# Supplementary material for: Effects of perspective switching and utilitarian thinking on moral judgments in a sacrificial dilemma among healthcare and non-healthcare students
Source: Curr Psychol. 2023 Feb 16:1–13. Online ahead of print. doi: 10.1007/s12144-023-04380-z (PMC9932409; doi:10.1007/s12144-023-04380-z)
Supplement: Supplementary file 1 — Supplementary file1 (DOCX 26 KB) [file 12144_2023_4380_MOESM1_ESM.docx]

**Appendix: “Effects of perspective switching and utilitarian thinking on moral judgments in sacrificial dilemma among healthcare and non-healthcare students”**

**Table S1. Oxford Utilitarianism Scale (Kahane et al., 2018)**

| **Impartial Beneficence** | |
| --- | --- |
| 1 | From a moral perspective, people should care about the well-being of all human beings on the planet equally; they should not favor the well-being of people who are especially close to them either physically or emotionally. |
| 2 | From a moral point of view, we should feel obliged to give one of our kidneys to a person with  kidney failure since we don’t need two kidneys to survive, but really only one to be healthy. |
| 3 | If the only way to save another person’s life during an emergency is to sacrifice one’s own leg, then one is morally required to make this sacrifice. |
| 4 | It is just as wrong to fail to help someone as it is to actively harm them yourself. |
| 5 | It is morally wrong to keep money that one doesn’t really need if one can donate it to causes that provide effective help to those who will benefit a great deal. |
| **Instrumental Harm** | |
| 1 | It is morally right to harm an innocent person if harming them is a necessary means to helping  several other innocent people. |
| 2 | If the only way to ensure the overall well-being and happiness of the people is through the use of political oppression for a short, limited period, then political oppression should be used. |
| 3 | It is permissible to torture an innocent person if this would be necessary to provide information to prevent a bomb going off that would kill hundreds of people. |
| 4 | Sometimes it is morally necessary for innocent people to die as collateral damage—if more people are saved overall. |

**Table S2. List of Modified Moral dilemmas (Moore et al., 2008)**

| ***Neutral* context** | |
| --- | --- |
| Crying baby | Enemy soldiers have taken over a village. They have orders to kill all remaining civilians over the age of two. Some of the townspeople have sought refuge in two rooms of the cellar of a large house. Outside the villagers hear the voices of soldiers who have come to search the house for valuables. A baby, who is with a parent amongst the villagers in the room, begins to cry loudly. The parent put his/her hand over the baby’s mouth to block the sound. If the parent removes his/her hand from the baby’s mouth the baby’s crying will summon the attention of the soldiers who will spare the baby’s life, but will kill the parent and the others hiding in both rooms. To save oneself and the others the parent must keep his/her hand on the baby’s mouth and smother the baby to death. Should the parent smother his/her baby in order to save oneself and the other townspeople?  Please choose whether you agree or disagree with the suggested action in the scenario.  Please select the extent to which you agree with your choices above. |
| Burning building | A total of seven people are trapped in a burning building. There is only one emergency exit through which everyone could escape to safety, but it is blocked by burning debris. One of the seven people in the hallway leading to the exit who has been injured is about to crawl to safety through a small hole at the bottom of the exit door. The other six people do not have time to climb through the small hole. The other six people could grab the injured person and use his/her body as a battering-ram to break through the burning blockage in the hallway that is preventing their escape. Doing this is certain to kill the injured person. However, if the rest of the people do not do this, they will die. Should the six people kill the injured person in order to save themselves?  Please choose whether you agree or disagree with the suggested action in the scenario.  Please select the extent to which you agree with your choices above. |
| Submarine | An onboard explosion has damaged a submarine, killed and injured several crewmembers. Additionally, it has collapsed the only access corridor between the upper and lower parts of the submarine. The upper section, where the most of the crewmembers are located, does not have enough oxygen remaining for all of the crewmembers to survive until they reach the surface. Only one remaining crewmember is located in the lower section, where there is enough oxygen. There is an emergency access hatch between the upper and lower sections of the submarine. If released, it will fall to the deck and allow oxygen to reach the upper section. However, a crewmember in the lower section was knocked unconscious and is lying beneath the hatch while the rest of the crew are almost out of air. If the crewmembers in the upper section shove the hatch open they will have air, but it will fall to the deck, crushing the unconscious crewmember to death. Should the crewmembers in the upper deck open the hatch and crush the crewmember below to save crewmembers in the upper deck?  Please choose whether you agree or disagree with the suggested action in the scenario.  Please select the extent to which you agree with your choices above. |
| ***Self-in-minority* context** | |
| Crying baby | Enemy soldiers have taken over a village. They have orders to kill all remaining civilians over the age of two. You and some of the townspeople have sought refuge in two rooms of the cellar of a large house. Outside you and the villagers hear the voices of soldiers who have come to search the house for valuables. Your baby, who is with you in the room, begins to cry loudly. You put your hand over the baby’s mouth to block the sound. If you remove your hand from the baby’s mouth the baby’s crying will summon the attention of the soldiers who will spare the baby’s life, but will kill you and the others hiding in both rooms. To save yourself and the others you must keep your hand on the baby’s mouth and smother the baby to death. Should you smother your baby in order to save yourself and the other townspeople?  Please choose whether you agree or disagree with the suggested action in the scenario.  Please select the extent to which you agree with your choices above. |
| Burning building | You and six other people are trapped in a burning building. There is only one emergency exit through which all of you could escape to safety, but it is blocked by burning debris. One of the seven people in the hallway leading to the exit who has been injured is about to crawl to safety through a small hole at the bottom of the exit door. The other six people do not have time to climb through the small hole. The other six people could grab you and use your body as a battering-ram to break through the burning blockage in the hallway that is preventing their escape. Doing this is certain to kill you. However, if the rest of the people do not do this, they will die. Should the six people kill you in order to save themselves?  Please choose whether you agree or disagree with the suggested action in the scenario.  Please select the extent to which you agree with your choices above. |
| Submarine | An onboard explosion has damaged a submarine, killed and injured several crewmembers. Additionally, it has collapsed the only access corridor between the upper and lower parts of the submarine. The upper section, where the most of the crewmembers are located, does not have enough oxygen remaining for all of the crewmembers to survive until they reach the surface. You are located in the lower section by yourself, where there is enough oxygen. There is an emergency access hatch between the upper and lower sections of the submarine. If released, it will fall to the deck and allow oxygen to reach the upper section. However, you were knocked unconscious and are lying beneath the hatch while the rest of the crew are almost out of air. If the crewmembers in the upper section shove the hatch open they will have air, but it will fall to the deck, crushing you to death. Should the crewmembers in the upper deck open the hatch and crush you below to save crewmembers in the upper deck?  Please choose whether you agree or disagree with the suggested action in the scenario.  Please select the extent to which you agree with your choices above. |
| ***Self-in-majority* context** | |
| Crying baby | Enemy soldiers have taken over a village. They have orders to kill all remaining civilians over the age of two. You and some of the townspeople have sought refuge in two rooms of the cellar of a large house. Outside you and the villagers hear the voices of soldiers who have come to search the house for valuables. A baby, who is with a villager in the room, begins to cry loudly. The parent put his/her hand over the baby’s mouth to block the sound. If the parent removes his/her hand from the baby’s mouth the baby’s crying will summon the attention of the soldiers who will spare the baby’s life, but will kill the parent and the others, including you, hiding in both rooms. To save yourself and the others the parent must keep his/her hand on the baby’s mouth and smother the baby to death. Should the parent smother his/her baby in order to save him/herself, you and the other townspeople?  Please choose whether you agree or disagree with the suggested action in the scenario.  Please select the extent to which you agree with your choices above. |
| Burning building | You and six other people are trapped in a burning building. There is only one emergency exit through which everyone could escape to safety, but it is blocked by burning debris. One of the seven people in the hallway leading to the exit who has been injured is about to crawl to safety through a small hole at the bottom of the exit door. You and the other five people do not have time to climb through the small hole. You and the other five people could grab the injured person and use his/her body as a battering-ram to break through the burning blockage in the hallway that is preventing their escape. Doing this is certain to kill the injured person. However, if you and the other five people do not do this, you and the other five people will die. Should you and the other five people kill the injured person in order to save yourself and the other five people?  Please choose whether you agree or disagree with the suggested action in the scenario.  Please select the extent to which you agree with your choices above. |

**Table S3. Control variables**

| **Empathic concern (Davis, 1983)** | |
| --- | --- |
| 1 | I often have tender, concerned feelings for people less fortunate than me. |
| 2 | Sometimes I don't feel very sorry for other people when they are having problems. (reversed-scored item) |
| 3 | Other people's misfortunes do not usually disturb me a great deal. (reversed-scored item) |
| 4 | When I see someone being treated unfairly, I sometimes don't feel very much pity for them. (reversed-scored item) |
| 5 | I am often quite touched by things that I see happen. |
| 6 | I would describe myself as a pretty soft-hearted person. |
| **Cognitive reappraisal (Gross and John, 2003)** | |
| 1 | When I want to feel more positive emotion (such as joy or amusement), I change what I’m thinking about. |
| 2 | When I want to feel less negative emotion (such as sadness or anger), I change what I’m thinking about. |
| 3 | When I’m faced with a stressful situation, I make myself think about it in a way that helps me stay calm |
| 4 | When I want to feel more positive emotion, I change the way I’m thinking about the situation. |
| 5 | I control my emotions by changing the way I think about the situation I’m in. |
| 6 | When I want to feel less negative emotion, I change the way I’m thinking about the situation. |

**Reference**

Davis, M. H. (1983). Measuring individual differences in empathy: Evidence for a multidimensional approach. *Journal of Personality and Social Psychology*, *44*(1), 113 – 126. https://doi.org/10.1037/0022-3514.44.1.113

Gross, J. J., & John, O. P. (2003). Individual differences in two emotion regulation processes: implications for affect, relationships, and well-being*. Journal of Personality and Social Psychology*, *85*(2), 348. https://doi.org/10.1037/0022-3514.85.2.348

Kahane, G., Everett, J. A. C., Earp, B. D., Caviola, L., Faber, N. S., Crockett, M. J., & Savulescu, J. (2018). Beyond sacrificial harm: A two-dimensional model of utilitarian psychology. *Psychological Review, 125*(2), 131–164. https://doi.org/10.1037/rev0000093

Moore, A. B., Clark, B. A., & Kane, M. J. (2008). Who Shalt Not Kill? Individual Differences in Working Memory Capacity, Executive Control, and Moral Judgment. *Psychological Science, 19*(6), 549–557. https://doi.org/10.1111/j.1467-9280.2008.02122.x
